# An Evaluation of Matrix-Containing and Humanised Matrix-Free 3-Dimensional Cell Culture Systems for Studying Breast Cancer

**DOI:** 10.1371/journal.pone.0157004

**Published:** 2016-06-14

**Authors:** Grace C. Roberts, Paul G. Morris, Marcus A. Moss, Sarah L. Maltby, Chelsea A. Palmer, Claire E. Nash, Emily Smart, Deborah L. Holliday, Valerie Speirs

**Affiliations:** Leeds Institute of Cancer and Pathology, Wellcome Trust Brenner Building, University of Leeds, Leeds, LS9 7TF, United Kingdom; Mayo Clinic College of Medicine, UNITED STATES

## Abstract

**Background:**

3D cell cultures are emerging as more physiologically meaningful alternatives to monolayer cultures for many biological applications. They are attractive because they more closely mimic in vivo morphology, especially when co-cultured with stromal fibroblasts.

**Methodology/Principal Findings:**

We compared the efficacy of 3 different 3D cell culture systems; collagen I, low attachment culture vessels and a modification of Fibrolife®, a specialised humanised cell culture medium devoid of animal-derived components, using breast cancer cell lines representative of the different molecular subtypes of breast cancer, cultured alone or with human mammary fibroblasts with a view to developing matrix-free humanised systems. 3D collagen I culture supported the growth of a range of breast cancer cell lines. By modifying the composition of Fibrolife® to epiFL, matrix-free cell culture was possible. During sequential transfer to epiFL breast cancer cells gradually detached from the flask, growing progressively as spheroids. Phenotype was stable and reversible with cells remaining actively proliferating and easily accessible throughout culture. They could also be revived from frozen stocks. To achieve co-culture with fibroblasts in epiFL required use of low attachment culture vessels instead of standard plastic as fibroblasts remained adherent in epiFL. Here, cancer cell spheroids were allowed to form before adding fibroblasts. Immunohistochemical examination showed fibroblasts scattered throughout the epithelial spheroid, not dissimilar to the relationship of tumour stroma in human breast cancer.

**Conclusions:**

Because of its ease of handling, matrix-free 3D cell culture may be a useful model to study the influence of fibroblasts on breast cancer epithelial cells with use of epiFL culture medium taking this a step further towards a fully humanised 3D model. This methodology could be applied to other types of cancer cell lines, making this a versatile technique for cancer researchers wishing to use *in vitro* systems that better reflect cancer *in vivo*.

## Introduction

Traditionally, cancer biologists have performed experiments in 2-dimensions (2D), with cells grown on plastic vessels designed to encourage cell adhesion. This adhesion forces the cells to artificially polarise even inducing polarity in some cell types, such as fibroblasts, which would not polarise *in vivo* [1]. However, pioneering work by Bissell and others have led to a gradual recognition that growing cells in 2D on plastic substrates is inadequate [2–7]. Furthermore, experiments comparing cells in 2D versus more physiologically relevant 3-dimensional (3D) cell culture systems have highlighted changes in cell morphology, behaviour and signalling pathways in comparison to 2D cell culture [8–12]. As a result, more emphasis is being placed on 3D culture systems, with over 900 original publications now on PubMed (date accessed 5 April 2016), though these studies often still rely on 2D techniques for maintenance of adherent cell lines.

Most current 3D cell culture models in routine use require a matrix. Many commercial matrices are available including collagen, Matrigel™ and other synthetic support systems [3, 4, 13–15]. These matrices allow cells to migrate and organise themselves into structures which are more representative of *in vivo* tissues, exemplified in particular in 3D models of normal mammary gland where reorganisation of cells into phenotypes reminiscent of the morphology of the normal gland is seen [16, 17], while breast cancer cells tend to form spheroids [18–20]. However commercially available matrices sometimes contain an array of growth factors which can alter cellular activity, allowing expansion of sub-populations which may enhance tumorigenesis [21]. Furthermore many 3D cell culture systems can be labour intensive to establish with experiments often taking weeks to reach a conclusion. Once cells are within a matrix it can sometimes be challenging to remove them easily at the same time retaining viability, which can limit the scope of the downstream experimental workflow. An ideal matrix-free 3D culture system would offer similar characteristics to those grown in matrices but without the disadvantages listed above.

A further consideration for scientists is to make their models systems as physiologically relevant as possible at the same time taking account of the 3Rs; Replacement, Reduction and Refinement, developed over 50 years ago as a basis for humane animal research and which now form national and international legislation governing the use of animals in scientific research. As such the ‘holy grail’ for cell culture scientists is to grow human cells under humanised conditions. In practice this would involve growing cells in well-defined humanised media systems under conditions which permit cells to develop a phenotype comparable to those observed *in vivo*. Standard cell culture media is normally supplemented with foetal calf serum (FCS) which is not without its ethical and scientific issues. The latter includes batch variability, risk of contamination from bacteria, viruses or mycoplasma and varying amount of growth factors, immunoglobulins and transcription factors which may impact on cell morphology and behaviour [22]. Over the past decade, chemically defined media have appeared on the markets that are entirely free of animal derived factors. These typically consist of a basic media supplemented with human growth factors including EGF, FGF, TGF-β, insulin and a protein component such as human serum or human serum albumin instead of FCS.

The aim of this work was to compare the efficacy of 3 different 3D cell culture systems; type I collagen, a modification of Fibrolife®, a specialised humanised cell culture medium [23], and low attachment culture vessels using a range of breast cancer cell lines cultured alone or with mammary fibroblasts with a view to developing a suitable humanised matrix-free culture for co-culture, providing scientists with a research tool to help begin to fulfil the 3Rs.

## Materials and Methods

### Cell culture

A panel of breast cancer cell lines were selected to represent the different molecular classes of breast cancer cells [24] including: MCF-7, T47D, MDA-MB-453, MDA-MB-468, MDA-MB-231 and BT474, as well as HFFF2 human foreskin fibroblasts, all obtained originally from American Type Culture Collection (Rockville, Maryland, US) and maintained under recommended culture conditions (see S1 Table). To avoid genetic drift early passage cell stocks were used for no more than 3 months, after which they were replaced with frozen stocks from the same original early passage stocks. Quarterly mycoplasma tests (MycoAlert, Lonza, Switzerland) were consistently negative and annual STR testing (Powerplex16, Promega, Madison, Wisconsin, US) confirmed cell identity. Following ethical approval (Leeds East Research Ethics Committee 09/H1036/108) and written consent from donors, primary human mammary fibroblasts were generated in house from normal breast tissue obtained from a reduction mammoplasty [25]. One of these, LS11-083, was hTERT-immortalised [26] and ds-Red transduced, to increase longevity and enable tracking in culture, as previously described [16]. Where measured, cell growth was estimated using a haemocytometer.

### 3D collagen culture

Breast cancer cells (3.3x10^5^) were resuspended in 2 ml of DMEM and aggregated over agar (2h at 37°C, 5% CO_2_). Aggregates were centrifuged at 500 rpm (3 min) and resuspended in 1 ml of rat tail collagen I (BD Biosciences, Franklin Lakes, New Jersey, US). This was adjusted to 1 mg/ml by diluting with Dulbecco’s PBS. Eight parts of collagen 1 were mixed with one part 10x Hank’s buffered salt solution (Sigma-Aldrich, St Louis, Missouri, US). Sodium hydroxide was added drop wise to neutralise the pH. One volume of DMEM was added and mixed into the collagen 1 solution by gentle pipetting. One hundred μl of the gel mixture was pipetted onto a 10mm Petri dish (MatTek Corp, Ashland, Massachusetts, US), left to solidify (10 min 37°C, 5% CO_2_) before covering with 1.5 ml complete DMEM. Gels were cultured for 7 days, with media replaced every 2–3 days.

### Culture in Fibrolife®

FibroLife® (FL; XCellR8 Distribution, Daresbury, Cheshire, UK) is a specialised humanised cell culture medium devoid of animal-derived components and developed for culture of fibroblasts. It comprises basal media and optional growth factors; HLL supplement (HSA 500μg/ml, linoleic acid 0.6μm, lecithin 0.6μg/ml), rHFGF (5ng/ml), rhEGF/TGFβ1 (5ng/ml, 30pg/ml), rhinsulin(5μg/ml), ascorbic acid (50μg/ml), L-glutamine (7.5mM) and hydrocortisone hemisuccinate (1μg/ml). LS11-083 human mammary fibroblasts were cultured in standard growth media (DMEM + 10% FCS) or in FL supplemented with the growth factors listed above. After 6 days cells were trypsinised and counted. Subsequently, FL was modified to make the formulation more similar to the breast cancer epithelial cell culture media by omission of FGF and rhEGF/TGF-β. This formulation was renamed epiFibrolife (epiFL). Breast cancer cell lines were sequentially passaged into increasing ratio of epiFL to normal maintenance media: 25:75, 50:50, 75:25 and then into 100% epiFL. Phase contrast images were taken daily to assess cell phenotype (Nikon, Tokyo, Japan). Once transfers were complete cells were maintained in T25 culture flasks (Corning, Corning, New York, US). epiFL was refreshed twice weekly by allowing the cells to sediment, removing 50% of the media volume and replacing with fresh media. If flasks became densely packed they were split 1:2 into a new flask. To determine if the phenotype was reversible, growth factors and serum were sequentially added back to cells growing in epiFL as follows: RPMI/5%FCS, epiFL + EGF/TGF-β, epiFL + EGF/TGFβ+FGF, epiFL + EGF/TGFβ+FGF +5% FCS) and effects on phenotype observed.

### Culture on low-attachment plates

MCF-7 cells were seeded at a density of 1 x 10^6^ cells into low-attachment T25 flasks or standard flasks (both Corning) in RPMI supplemented with 5% FCS. At 48h, LS11-083 human mammary fibroblasts were added. Cells were imaged directly in culture using a dual purpose phase contrast and fluorescence microscope (EVOS FL Cell Imaging System, Life Technologies, Paisley, UK), for up to 96h.

### Viability testing

Cell viability in 2D and 3D was quantified using a ViCell (Beckman Coulter, Brea, California, US). Cells in 2D were trypsinised (5 minutes, 37°C) before resuspension in media. Cells in 3D were pelleted (500 rpm, 3 minutes), media removed and cells resuspended in 1 mL trypsin and incubated for 5 minutes to facilitate spheroid dispersal. Cells were then resuspended in media and then applied to the ViCell.

### Preparation of spheroids for immunohistochemistry, Picro-Sirius Red histochemistry and immunofluorescence

Spheroids resulting from culture in epiFL and low attachment plates were carefully transferred to 60 ml centrifuge tubes (Corning) containing 50 ml of 10% formalin (Sigma) and fixed overnight before rinsing (PBS). They were then transferred to a water bath (50°C), 1.5% agarose solution was added and incubated for 30–60 minutes. Tubes were then removed and placed on ice to allow the agarose to set. The pellet was removed from the tube, processed (Leica ASP200 processor, Leica, Wetzlar, Germany) and set in a paraffin block. Five μm serial or semi-serial sections were cut from these blocks (Leica 2030 microtome,) onto SuperFrost plus+ slides (ThermoScientific, Waltham, Massachusetts, US). Slides were dried overnight (37°C incubator). Prior to immunohistochemistry, slides were baked on a hot plate (70°C, 1 h), then pressure cooked (125°C, 2 minutes) in antigen retrieval solution. Slides were transferred to hot tris-borate saline buffer and run under tap water until cool. The Leica Novolink Polymer Detection System was used as per manufacturer’s specifications for Ki67 (M7240), E-cadherin (M3612) and Vimentin (M7020, all Dako, Glostrup, Denmark) and M30 (12140322001, Roche, Basel, Switzerland). For M30, slides were de-waxed in xylene and ethanol prior to antigen retrieval. Hydrogen peroxidase (Sigma) was used as a peroxidase block and Casein (Vector, Burlingame, California, US) as a protein block. For Picro-Sirius Red histochemistry, sections were dewaxed through xylene then stained with Weigert’s Haematoxylin (15 minutes) and differentiated in 1% acid alcohol. Collagen was then visualised using 0.1% Picro-Sirius Red solution for 1 hour. Slides were dehydrated through graded ethanol and cleared in xylene. All stained slides were scanned at x20 (Aperio XT, Leica). Co-cultures were also imaged directly in culture using a dual purpose phase contrast and fluorescence microscope (EVOS FL Cell Imaging System, Life Technologies, Paisley, UK)).

### Statistical analysis

Two-tailed Students’ t-test was performed (GraphPrism, GraphPad Software, Inc. La Holla, CA, US) to determine differences in cell growth in standard culture medium or FL. A P value < 0.05 was considered significant.

## Results

### Growth in 3D collagen

**Fig 1:**
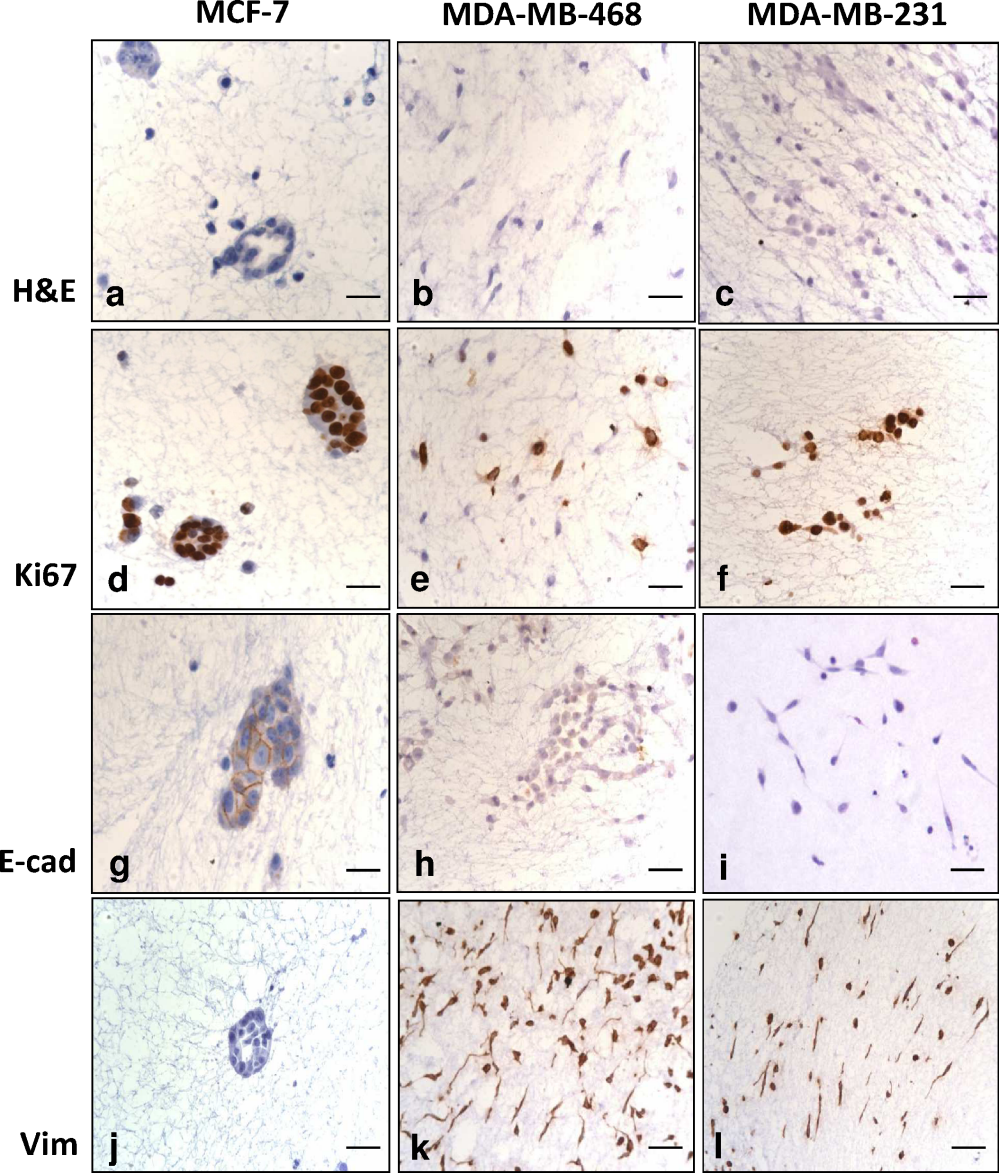
Morphological appearance of breast cancer cell lines cultured in 3D collagen gels for 7 days. By H&E, MCF-7 (a) cells formed tight spheroids while MDA-MB-468 (b) and MDA-231 (c) cells did not. All 3 cell lines displayed select Ki67-positive nuclei (d-f). E-cadherin expression was observed in MCF-7 cells (g), but not MDA-MB-468 (h) or MDA-231 (i) cells while vimentin expression was lacking in MCF-7 cells (j), but expressed in MDA-MB-468 (k) and MDA-MB-231 cells (l). Scale bar = 80μm.

Comparison of luminal A (MCF-7), basal (MDA-MB-453) and basal/claudin-low (MDA-MB-231) cell lines showed luminal A cells formed roughly spherical masses in 3D collagen. In contrast MDA-MB-231 and MDA-MB-453 cells were scattered and discohesive. All cells types examined displayed select Ki67-positive nuclei. E-cadherin was restricted to MCF-7 cells with vimentin was observed in MDA-MB-231 and MDA-MB-453 cells. Representative examples are shown in Fig 1.

### Fibroblast growth in FL

**Fig 2:**
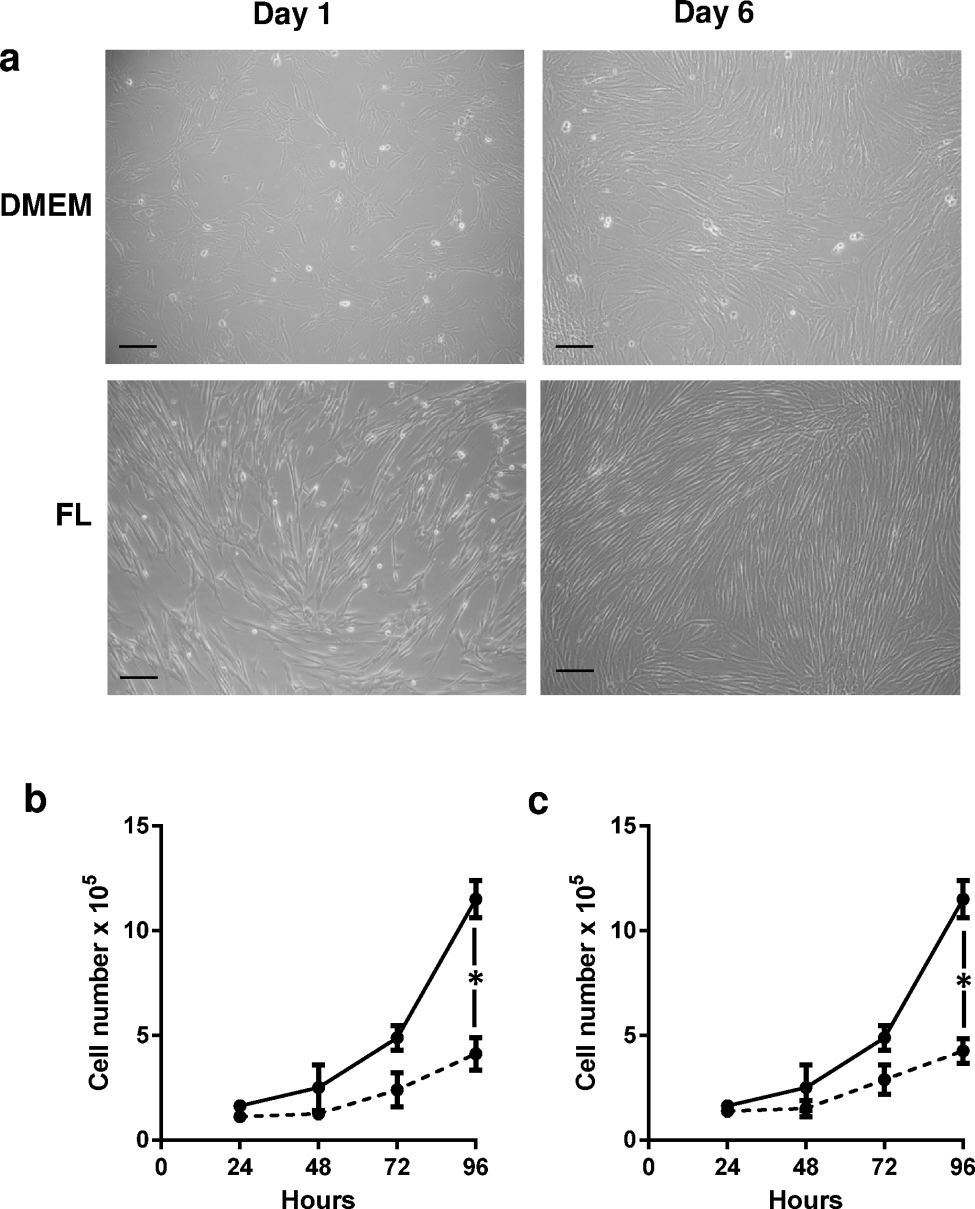
Phenotypically, when viewed under phase contrast microscopy, human mammary fibroblasts have greater terminal cell density when cultured in FL medium compared to DMEM supplemented with 10% FCS (a). This was confirmed by direct cell counting of HFFF2 fibroblasts cultured in both types of media (mean ± SEM, n = 3;b). Scale bar = 100μm.

As FL was developed to support the growth of fibroblasts we assessed this first. No notable differences in cell morphology were observed between primary normal mammary fibroblasts or in HFFF2 fibroblasts grown in their standard medium or in FL. Fibroblasts demonstrated their classical elongated, spindle-like appearance (Fig 2A). After 6 days in culture HFFF2 fibroblasts grown in FL had significantly more cells, also reflected in human primary mammary fibroblasts (Fig 2B). However culture in FL did not prolong the lifespan of these primary fibroblasts; by passage 6 cells stopped proliferating, irrespective of the type of culture media.

### Effects of epiFL on breast cancer cell phenotype

**Fig 3:**
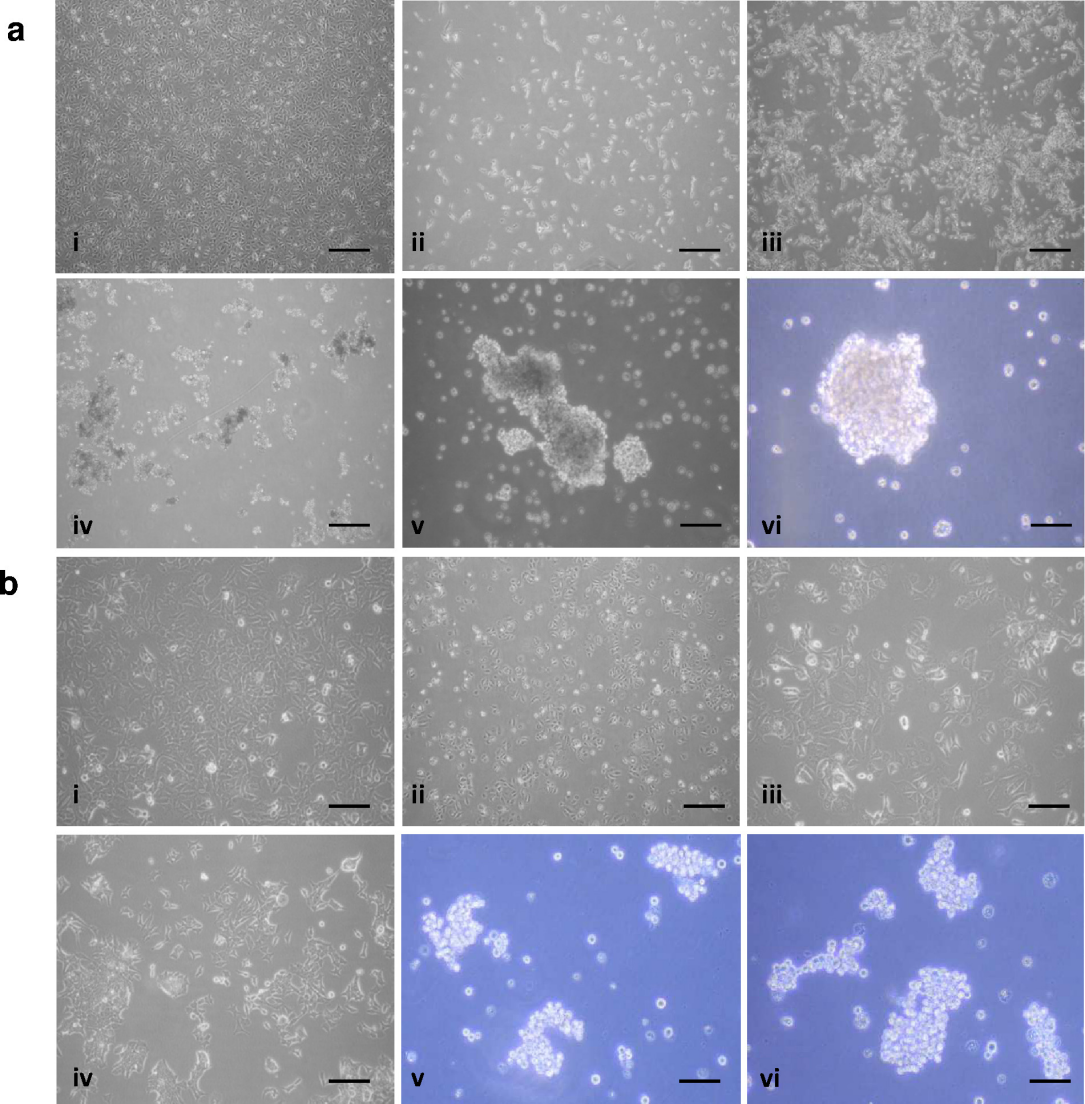
Sequentially increasing the amount epiFL in the culture medium of MCF- 7 (a) and MDA-MB-453 (b) cells resulted in a gradual progression from adherent monolayers (ai, bi) to floating cells (aii-iv; bii-iv) which formed tight spheroids (MCF-7) or grape-like clusters (MDA-MB-453) as culture conditions reached 100% epiFL (av-vi; bv-vi). Scale bar = 100μm.

epiFL was generated as described in the methods. When grown in their standard medium MCF-7 cells formed an adherent monolayer with classic cobblestone morphology associated with luminal epithelial cells (Fig 3Ai). During sequential transfer to epiFL the cells gradually detached from the flask, growing initially as single cells or small loosely-adherent clusters (Fig 3Aii–3Aiv) and gradually formed larger tighter spheroids which was most evident when transferred into 100% epiFL (Fig 3Av and 3Avi). This transition was also observed in MDA-MB-453 cells, although these cells formed much looser grape-like clusters (Fig 3Bi–3Bvi).

### Morphology and phenotype characterisation of breast cancer cells transferred to epiFL

**Fig 4:**
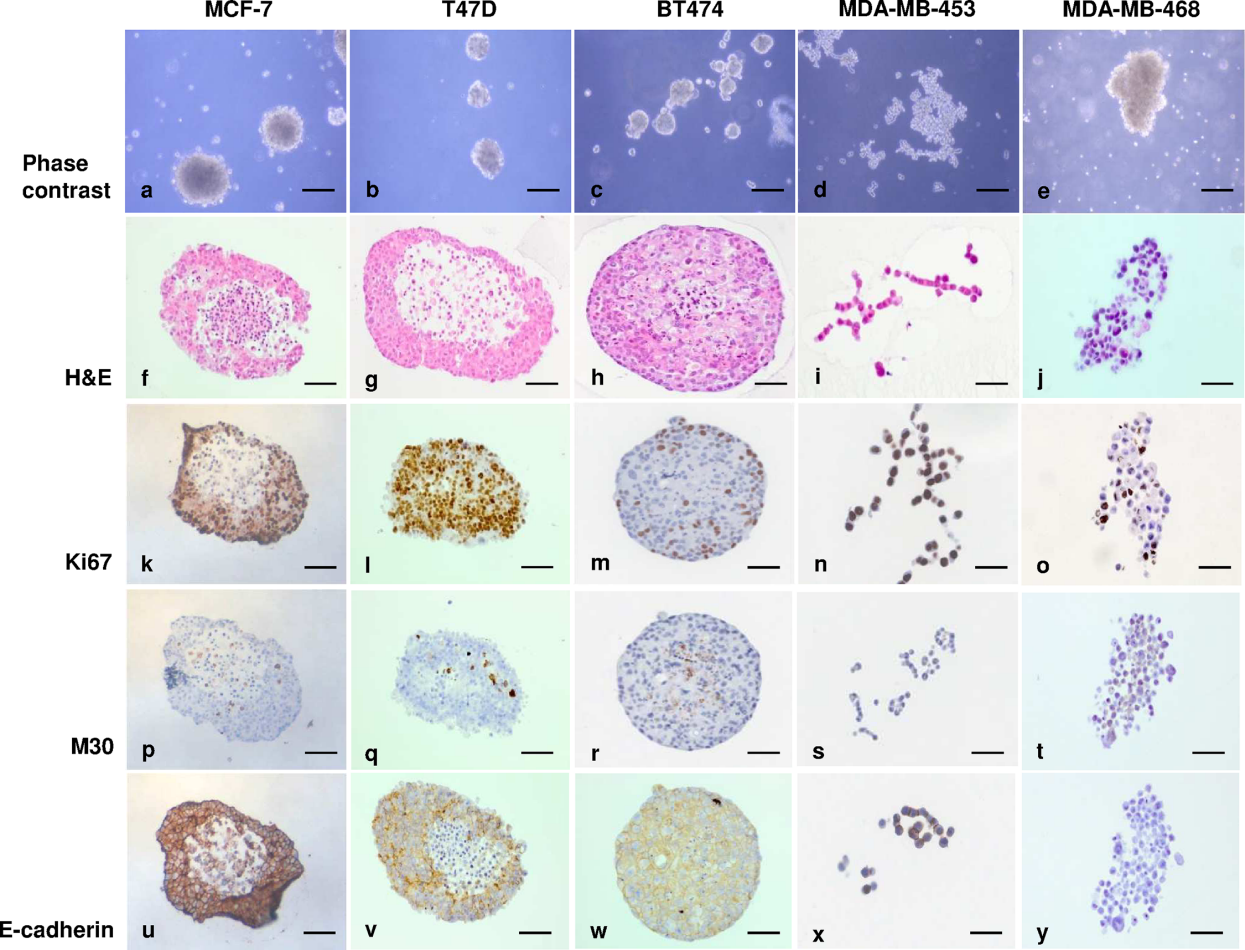
When cultured in epiFL, luminal A and B breast cancer cells lines grew as tightly packed spheroids (a-c), while HER-2 positive MDA-MB-453 cells (d) and basal MDA-MB-468 cells (e) formed loose aggregates. This phenotype was confirmed by H&E staining of formalin-fixed paraffin-embedded cell blocks (f-j). In luminal A and B cell lines, peripheral Ki67 immunoreactivity was observed (k, l, m) with areas of central apoptosis (p, q, r) and E-cadherin expression at areas of cell contact (u, v, w) While MDA-MB-468 and MDA-MB-453 cells expressed Ki67 (n, o) and some M30, a marker of apoptosis (s, t), E-cadherin was seen at areas of cell-cell contact in MDA-MB-453 but not MDA-MB-468 cells (x, y, respectively). Scale bar = 50μm.

As we were testing a new type of culture condition, we used an expanded cell line panel to examine the effect of culture in epiFL. Breast cancer cell lines representing different molecular subtypes were sequentially transferred from their standard culture medium (see S1 Table) into epiFL. Once established in 100% epiFL, cell morphology was assessed by phase contrast microscopy and phenotype by immunohistochemistry. The luminal A MCF-7 and T47D cells and luminal B BT474 cells formed dense spheroids when grown in epiFL (Fig 4A, 4B and 4C) while HER-2 positive MDA-MB-453 cells (Fig 4D) and basal MDA-MB-468 cells (Fig 4E) formed loose aggregates which could be partially disrupted with gentle agitation. The basal/claudin low MDA-MB-231 cells which grow on plastic as elongated spindly cells were unable to grow in epiFL.

H&E staining confirmed the tightly cohesive structures formed by T47D, MCF-7 and BT474 cells (Fig 4F, 4G and 4H). Ki67 immunoreactivity was observed (Fig 4K, 4L and 4M), mostly towards the spheroid periphery, with areas of central apoptosis delineated by M30 immunoreactivity (Fig 4P, 4Q and 4R). E-cadherin expression was seen in areas of cell contact (Fig 4U, 4V and 4W). The loose aggregates formed by MDA-MB-468 and MDA-MB-453 cells (Fig 4I and 4J) expressed Ki67 (Fig 4N and 4O) and some M30 (Fig 4S and 4T). E-cadherin was seen at areas of cell-cell contacts in MDA-MB-453 but not MDA-MB-468 cells (Fig 4X and 4Y, respectively). As the goal of our work was to develop a humanised matrix-free system suitable for the co-culture of breast epithelial cells with fibroblasts, we added epiFL medium to fibroblasts; cells remained adherent under these conditions, although they were less numerous with a stubby appearance (S1 Fig).

### Reversibility and stability of phenotype

**Fig 5:**
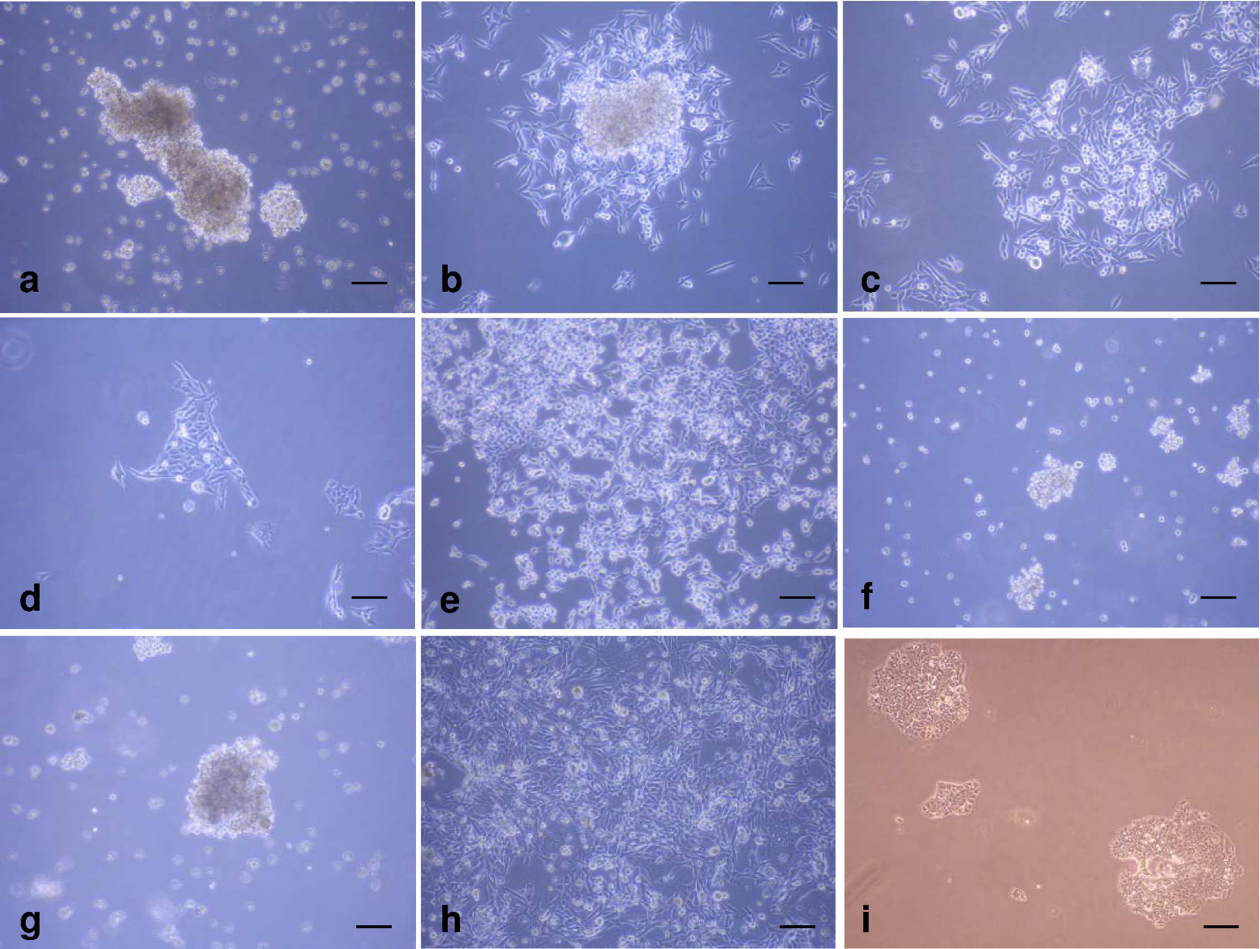
Effects of sequential transfer of MDA-MB-453 spheroids from epiFL back to their standard culture medium. Spheroids in epiFL (a). After 24 hours in 75% epiFL:25% RPMI + 5% FCS spheroids began to reattach (b). Sequential passage to 50% epiFL:50% RPMI + 5% FCS to 25% epiFL:75% RPMI + 5% FCS to 100% RPMI + 5% FCS cells resulted in monolayer formation (c-e, respectively). Re-adhesion could not be replicated by adding EGF/TGF (f) or EGF/TGF/FGF (g) into the epiFL but was achieved by adding 5% FCS to epiFL (Fig 5h). Directly replacing epiFL with standard culture medium resulted in cell reattachment to the culture flask and monolayer growth within 24 hours (i). Scale bar = 100μm.

Next we tested whether the phenotype was reversible using MDA-MB-453 and MCF-7 cells. MDA-MB-453 cells growing as spheroids in epiFL (Fig 5A) were sequentially transferred back into their standard culture medium. After 24 hours in 75% epiFL:25% RPMI + 5% FCS spheroids began to attach to the cell culture plates (Fig 5B). Through further passages to 100% RPMI + 5% FCS cells formed a complete monolayer with cell morphology comparable to MDA-MB-453 cells before epiFL culture. However cell re-adhesion could not be replicated by adding EGF/TGF (Fig 5F), or EGF/TGF/FGF (Fig 5G) into the epiFL but was achieved when 5% FCS was added to epiFL (Fig 5H). Directly replacing epiFL with standard culture media, resulted in cell reattachment to the culture flask and growth as a monolayer resumed within 24 hours (Fig 5I). Frozen stocks of cell cultures adapted to grow in epiFL were made and cells could be successfully revived after several months indicating stability of the phenotype. Using the same process, phenotypic reversion was also successfully achieved for MCF-7 cells (S2 Fig).

### Matrix-free co-culture of breast cancer cells with stromal fibroblasts in 3D

**Fig 6:**
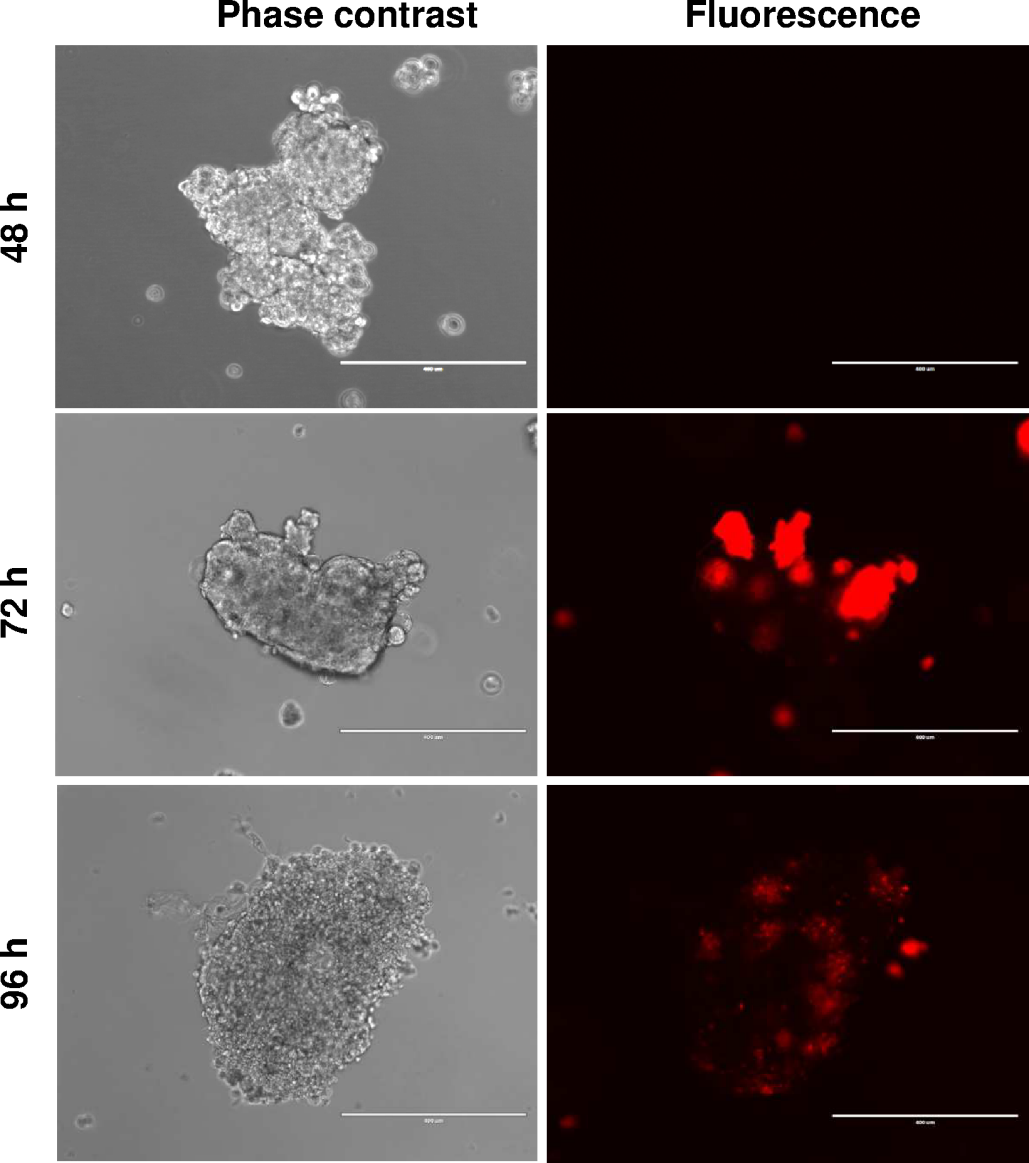
MCF-7 cells were plated on low adhesion plates and left for 48h undisturbed until spheroids formed. hTERT-immortalised fibroblasts transduced with dsRED, were then added and cocultured for a further 48h. Cells were imaged using a dual purpose phase contrast and fluorescence microscope, which allowed visualisation of cell distribution, defined by the fibroblasts’ red fluorescence and the MCF-7 cells’ lack of fluorescence under phase contrast. Scale bar = 400μm.

**Fig 7:**
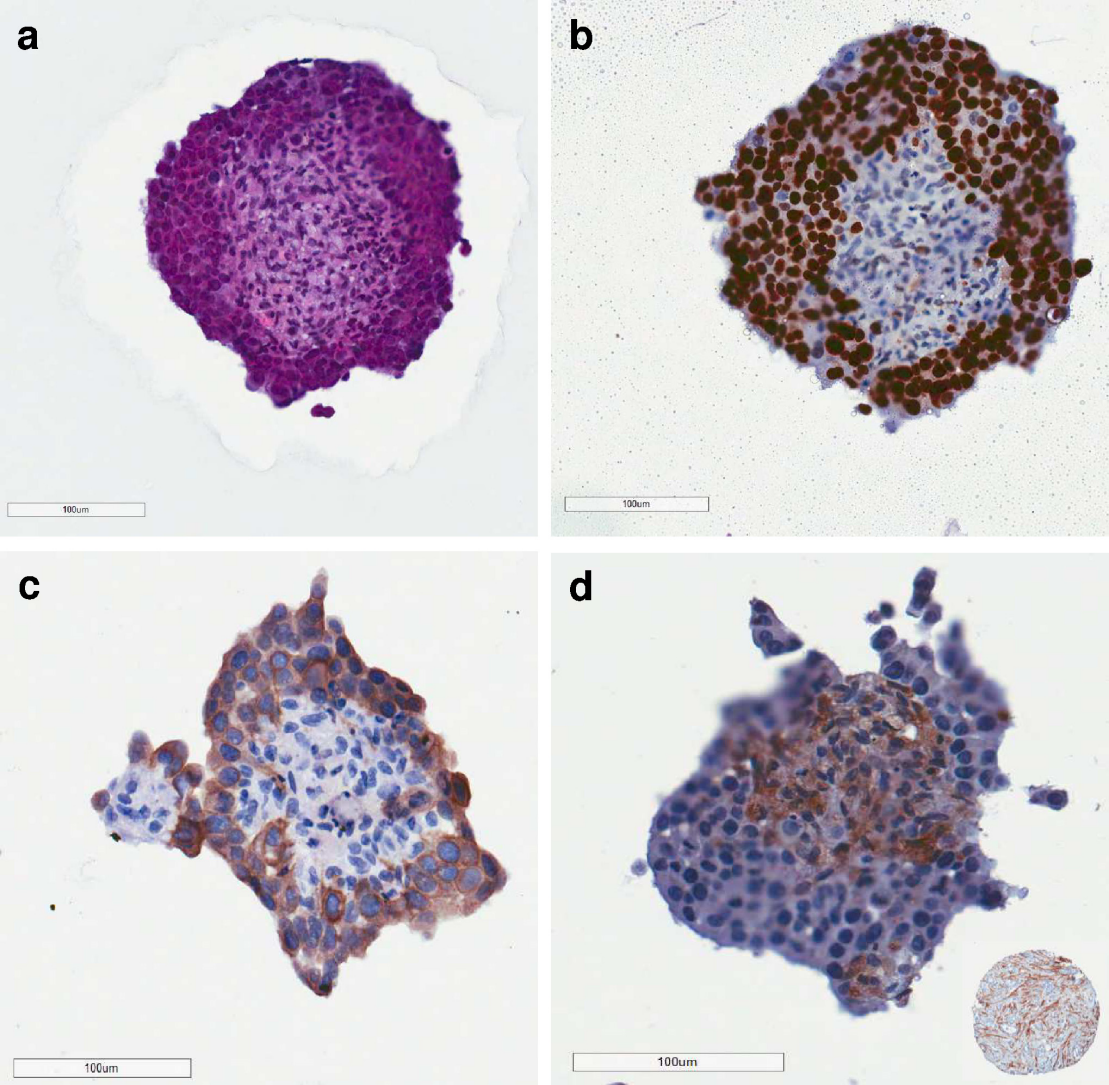
Immunohistochemical analysis of semi-serial sections of spheroid co-cultures. H&E staining of a representtaive spheroid is shown in (a). In (b) peripheral nuclear Ki67 immunoreactivity is seen, which seems to colocalise with epithelial cells, distinguished by cytokeratin 18 immunoreactivity, in an adjacent semi-serial section (c). Staining of a further semi-serial section with vimentin, used to detect fibroblasts shows fibroblast pockets scattered throughout the spheroid (d), not dissimilar to the relationship of tumour stroma in human breast cancer shown in the insert of (d), which illustrates a tissue microarray core stained with αSMA-stained stromal fibroblasts [44]. Positive immunoreactivity for each of the biomarkers is indicated by the brown staining. Scale bars = 100μm.

With increasing interest in the role of stromal fibroblasts in influencing cancer biology we examined if we could incorporate fibroblasts into our humanised matrix-free 3D culture model. It was not possible to achieve this in epiFL media as fibroblasts adhered to the culture vessel, in the same way they did when cultured in FL. However, co-culture of fibroblasts with MCF-7 cells was possible using low attachment culture vessels. A staggered co-culture was required by allowing MCF-7 spheroids to form first. After 48 hours, fibroblasts were added directly to the MCF-7 spheroids. Under these conditions, pockets of fibroblasts were visible, either attached to or within MCF-7 spheroids at 72 hours (i.e. 24 hours after adding them to the spheroids) under fluorescence microscopy, gradually dispersing within the MCF-7 spheroid after a futher 24 hours in culture (Fig 6). As illustrated in Fig 7, more detailed immunohistochemical analysis of serial/semi- serial sections prepared from fixed co-culture spheroids using epithelial and fibroblast-specific markers, showed that fibroblasts seemed to be surrounded by epithelial cells, a phenotype not dissimilar to the relationship of stromal fibroblasts within breast tumours, illustrated by the breast cancer TMA core, shown as an insert to Fig 7D, where stromal fibroblasts were identified by α-SMA staining. Differences in size between these formalin-fixed paraffin embedded images and the phase contrast/fluorescence images displayed in Fig 6 is most likely a result of fixation as formalin cross links proteins, which causes tissue shrinkage. Furthermore, evidence for collagen production was observed in the co-cultures, evidenced by a Picro Sirius red histochemistry (S3 Fig).

## Discussion

We have compared 3 methods of 3D cell culture using breast cancer cell lines as exemplars. Each model has strengths and weaknesses, allowing them to be applied in various fields of breast cancer research and to begin to work towards the development of fully humanised matrix-free 3D cell culture systems. Furthermore, the methodology we have employed could be applied to other types of cancer cell lines, making this a versatile technique for cancer researchers wishing to use *in vitro* systems that better reflect cancer *in vivo*.

Culture of normal breast and breast cancer cells in 3D collagen has been used successfully for many years [3–5, 9, 13, 16, 27–31] and we were able to replicate this in our work. As anticipated, cancer cells did not form lumens but instead formed solid or loosely adherent structures, reminiscent of the pioneering work of the Bissell laboratory [9]. A potential drawback of using cells embedded within a matrix is that cells can sometimes be difficult to remove for subsequent downstream analysis, hence we turned to matrix-free 3D culture. We were also mindful of working towards using a humanised system. This is attractive to scientists as it moves the experimental workflow a step closer to the *in vivo* state, at the same time addressing the 3Rs.

We were able to generate 3D cultures using 2 matrix-free cell culture systems; epiFL, low attachment plastic and, working towards a more humanised system, a combination of both. Matrix-free culture has many benefits over matrix-containing systems. Spheroids formed within a few days while this can often take up to 3 weeks in collagen. Because they are not embedded within a matrix, under matrix-free 3D, spheroids can be examined intact or easily dispersed from spheroids as single cells by gentle pipetting if required for further experimental use. Moreover, matrix-free systems are relatively inexpensive in comparison to matrix-containing systems, which may be an important consideration for some lab budgets.

FL medium was originally developed for the culture of fibroblasts and promotes fibrocyte differentiation from human and murine CD14+ peripheral blood monocytes [23]. We were able to demonstrate enhanced growth of fibroblasts in FL compared to DMEM, although this was unable to extend the lifespan of cultured primary fibroblasts. Adaptation of FL to epiFL permitted successful growth as 3D spheroids in 5 of the 6 breast cancer cell lines that we examined with only MDA-MB-231 cells unable to survive. Interestingly this cell line did grow in 3D on low attachment plates and our on-going experiments showed they appear to tolerate a subsequent change to epiFL. There is evidence from other work of diversity in the ability of cell lines to form 3D spheroids [32] [33] [34]. Some groups have shown MDA-MB-231 cells form only loose aggregates in 3D, described as a stellate phenotype [9], while others have shown spheroid formation in a variety of contexts [34, 35]. Moreover it is noteworthy from other work that MDA-MB-231 only formed tight aggregates when Matrigel™ was added to the cell suspension at the time of seeding [33].

Cells, once transferred into epiFL were stable and viable in culture for several months and the phenotype could be reversed by adding back their regular culture medium. Frozen stocks of these cultures were made and cells were successfully revived after several months. Morphology of these breast cancer cell lines in 3D differed with luminal-like cell lines T47-D, MCF-7 and BT474 cells forming tight spheroid structures and basal MDA-MB-468 and HER-2 positive MDA-MB-453 cells forming loosely cohesive aggregates. Similar morphologies have been observed when these cell types were cultured in 3D Matrigel™ which described round, mass, grape-like and stellate structures [9]. Under matrix-free conditions, our cell lines formed comparable mass and grape-like structures suggesting that the cells were behaving as if they were growing in a 3D matrix.

The 3D spheroids exhibited proliferation at the periphery with a central region of apoptosis. Whether this is a result of lack of nutrients or oxygen in the centre of the mass or loss of an environmental cue is unclear however, as the same picture was observed in smaller structures, it suggests both scenarios may be possible. With diffusion generally limited to ~100μm, the areas of apoptosis seen in the larger spheroids generated by the luminal cell lines (MCF-7, T47D and BT474) are more likely to be due to limitations in nutrient diffusion, although it should be noted that MCF-7 cells are resistant to apoptosis due to their deficiency in caspase 3 [36, 37]. Cell death in the centre of a tumour mass is a relatively common finding in human breast cancer particularly in ductal carcinoma *in situ* [38] highlighting similarities between our 3D spheroids and clinical breast cancer. The looser structures formed by MDA-MB-468 and MDA-MB-453 cells not surprisingly expressed less e-cadherin, which is a feature of these cells in vitro [39].

Despite advantages in using epiFL for matrix-free 3D culture, one of the initial drawbacks was the inability to use this in a co-culture model with fibroblasts. As these cells are now firmly recognised to influence tumour epithelial cells, having an easiier way to address this experimentally, particularly in terms of downstream measurements, is attractive to scientists. In our intial experiments, fibroblasts remained adherent in both FL and epiFL media. However, we overcame this by developing 3D spheroids growing in epiFL in low attachment culture vessels first, followed by subsequent addition of fibroblasts which became incorporated into these 3D spheroids after 24-48h. Fluorescence microscopy showed fibroblasts dotted within the 3D spheroids. Interestingly, more detailed immunohistochemical analysis of sections of these co-cultures using epithelial and fibroblast-specific markers showed that fibroblasts seemed to be surrounded by epithelial cells, a phenotype not dissimilar to the relationship of stromal fibroblasts within breast tumours.

We recognise a potential concern in using matrix-free culture is the lack of an extracellular matrix, often required to induce polarity. Both natural and synthetic matrices have been shown to do this, particularly in breast cancer [16, 40–42]. However, in our hands this did not appear to be an issue as Picro Sirius red, a histochemical stain which detects collagen [43] showed a pale red intercellular blush in our matrix-free co-cultures, which is characteristic of collagen deposition.

A potential weakness of our work is the absence of functional assays such as those addressing proliferation, invasion and migration. Nevertheless spheroids grown in 3D collagen and under matrix-free conditions in epi-FL both expressed E-cadherin, a calcium-dependent adhesion molecule which is found in epithelial cells. Our main motivation was to identify a system which is free of all non-human animal components, providing scientists with a potential ready to use solution to address the principles of the 3Rs: Replacement, Reduction and Refinement in their work, allowing functional assays to be explored in future. In our experiments with epiFL, we believe we have achieved this goal.

### Conclusions

Matrix-free 3D cell culture offers a useful model to study the influence of fibroblasts on breast cancer epithelial cells. Furthermore ease of access to cells throughout the culture period is a benefit over matrix-containing 3D culture where cells are generally inaccessible until the end of the experiment at which point it can sometimes be challenging to remove them for further downstream analysis. Finally the ability of breast cancer cells to grow in suspension in epiFL is a step towards developing a human only and cost effective cell culture method.

## Supporting Information

S1 Fig Phenotype of fibroblasts cultured in DMEM plus 10% FCS (a), FL (b) or epiFL (c). Cells remained adherent in all 3 different media formulations; those in epiFL appeared to lose the characteristic whorled phenotype seen in (a) and (b), displaying a more stubby appearance. Scale bar = 100μm.
(PDF)

S2 Fig Effects of transfer of MCF-7 spheroids from epiFL back to their standard culture medium. Spheroids in epiFL (a). After 24 hours in 75% epiFL:25% RPMI + 5% FCS spheroids began detach and adhere to the culture vessel (b) which was more pronounced after a further 24 hours in 50% epiFL:50% RPMI + 5% FCS (c) with an epithelial monolayer obtained once cells were restored to their native culture medium (100% RPMI + 5% FCS). Scale bar = 400μm.
(PDF)

S3 Fig Picro Sirius red histochemistry identifies collagen in a section of human breast tissue (a) shown by asterisks. Original magnification = 20x. Using the same histochemical stain there is evidence of collagen deposition in matrix-free 3D co-culture, illustrated by the consistent pink blush observed between the cells. Scale bar = 100μm.
(PDF)

S1 Table Culture conditions for the cells used in this study. (DOCX)

## Abbreviations

3Rs: Replacement, Reduction, Refinement
epiFL: epithelial Fibrolife®
FCS: foetal calf serum
FL: Fibrolife™
