# Supplementary figures and images for: An Evaluation of Matrix-Containing and Humanised Matrix-Free 3-Dimensional Cell Culture Systems for Studying Breast Cancer
Source: PLoS One. 2016 Jun 14;11(6):e0157004. doi: 10.1371/journal.pone.0157004 (PMC4907459; doi:10.1371/journal.pone.0157004)

**Fig S1**

**Phenotype of fibroblasts cultured in different media**

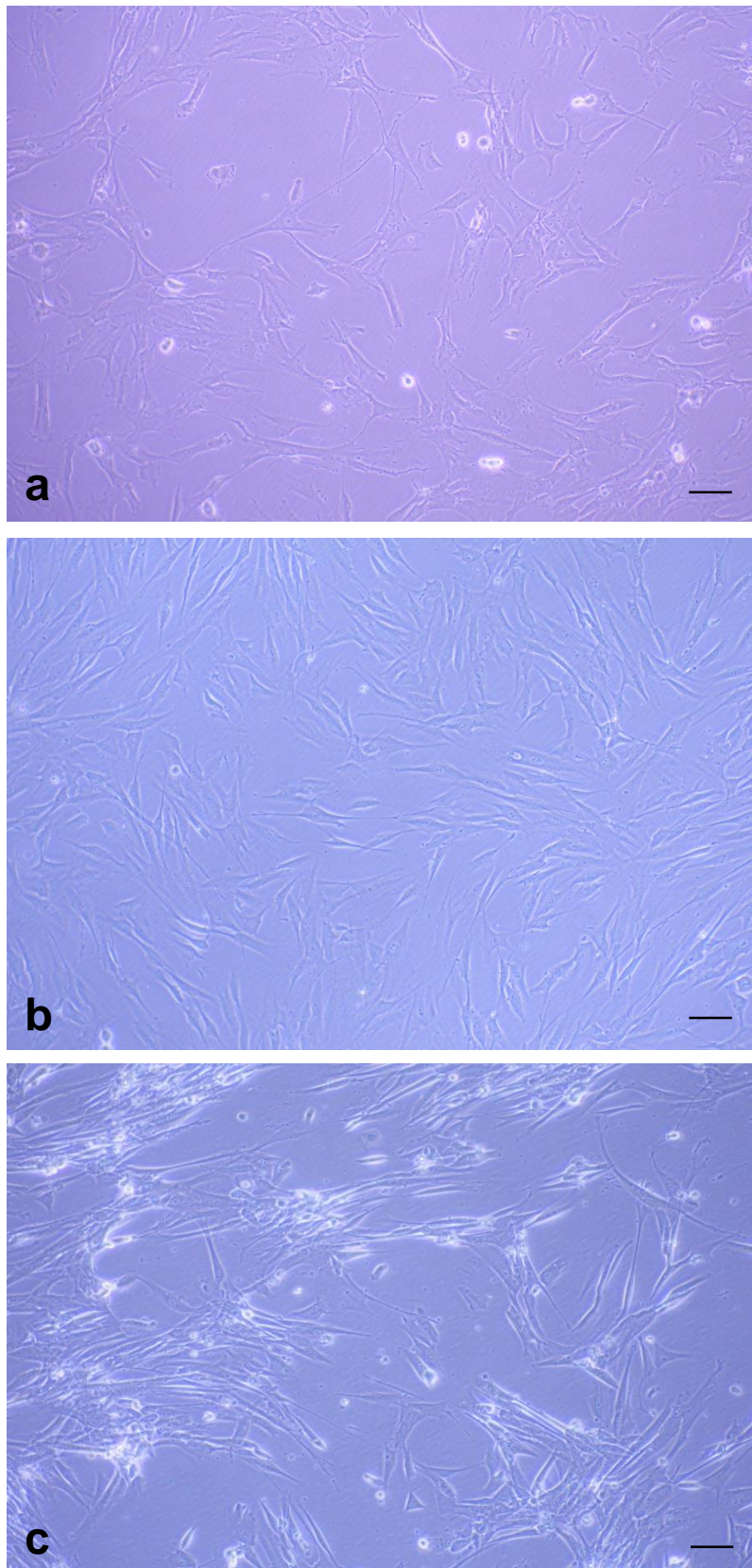

Supplement: S1 Fig — Phenotype of fibroblasts cultured in DMEM plus 10% FCS (a), FL (b) or epiFL (c). Cells remained adherent in all 3 different media formulations; those in epiFL appeared to lose the characteristic whorled phenotype seen in (a) and (b), displaying a more stubby appearance. Scale bar = 100μm. (PDF) [file pone.0157004.s001.pdf]

**Fig S2**

**Effects of transfer of MCF-7 spheroids from epiFL back to their standard culture medium**

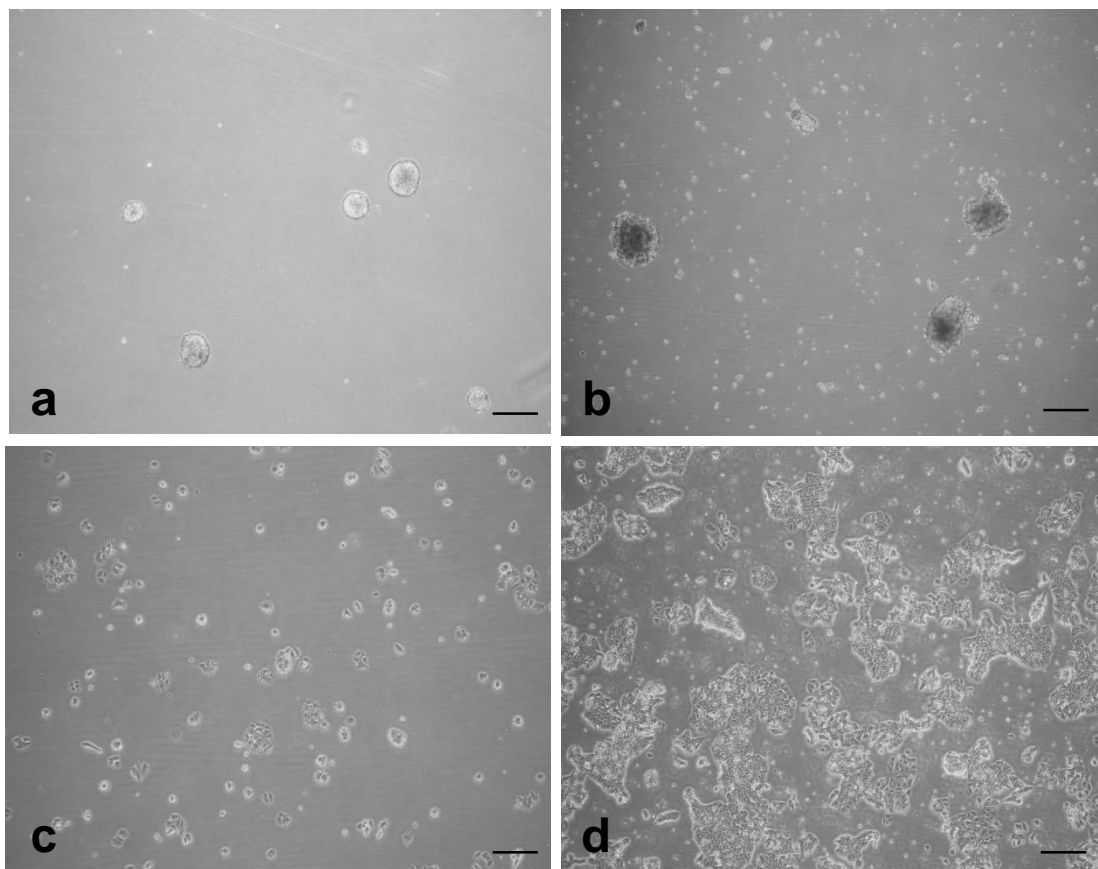

Supplement: S2 Fig — Spheroids in epiFL (a). After 24 hours in 75% epiFL:25% RPMI + 5% FCS spheroids began detach and adhere to the culture vessel (b) which was more pronounced after a further 24 hours in 50% epiFL:50% RPMI + 5% FCS (c) with an epithelial monolayer obtained once cells were restored to their native culture medium (100% RPMI + 5% FCS). Scale bar = 400μm. (PDF) [file pone.0157004.s002.pdf]

**Fig S3**  
**Picro Sirius red histochemistry**

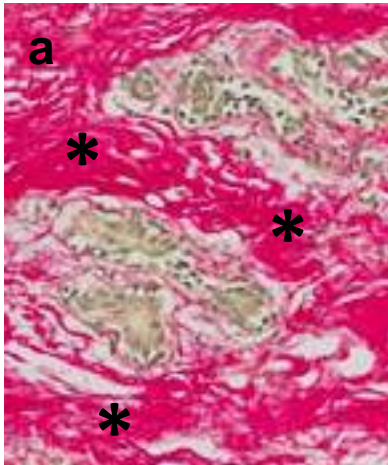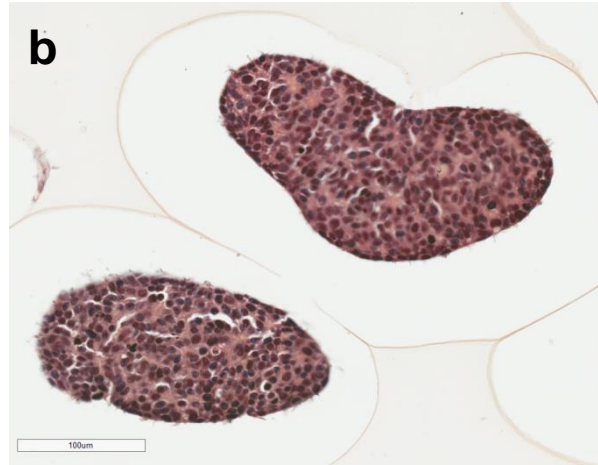

Supplement: S3 Fig — Picro Sirius red histochemistry identifies collagen in a section of human breast tissue (a) shown by asterisks. Original magnification = 20x. Using the same histochemical stain there is evidence of collagen deposition in matrix-free 3D co-culture, illustrated by the consistent pink blush observed between the cells. Scale bar = 100μm. (PDF) [file pone.0157004.s003.pdf]
