# Supplementary material for: An Evaluation of Matrix-Containing and Humanised Matrix-Free 3-Dimensional Cell Culture Systems for Studying Breast Cancer
Source: PLoS One. 2016 Jun 14;11(6):e0157004. doi: 10.1371/journal.pone.0157004 (PMC4907459; doi:10.1371/journal.pone.0157004)
Supplement: S1 Table — (DOCX) [file pone.0157004.s004.docx]

**Table S1**

**Culture conditions for the cells used in this study**

| **Cell Line** | **Classification^1^** | **Culture Medium^2^** |
| --- | --- | --- |
| MCF-7 | Luminal A | RPMI 1640 + 5% FCS |
| T47D | Luminal A | RPMI 1640 + 5% FCS |
| BT-474 | Luminal B | DMEM (glutamax)+ 10% FCS |
| MDA-MB-231 | Claudin-Low | RPMI 1640 + 5% FCS |
| MDA-MB-453 | Claudin-Low | RPMI 1640 + 10% FCS |
| MDA-MB-468 | Basal | RPMI 1640 + 5% FCS |
| BT-20 | Basal | RPMI 1640 + 10% FCS |
| MDA-MB-453 | Claudin-Low | RPMI 1640 + 10% FCS |
| SK-BR-3 | HER2 | DMEM (glutamax)+ 10% FCS |
| HFFF2 | Human fetal foreskin fibroblast | DMEM (glutamax)+ 10% FCS |
| LS11-083^3^ | Human mammary fibroblast, hTERT-immortalised | DMEM (glutamax)+ 10% FCS |

^1^Classification of the breast cancer cell lines was defined according to the following references: Neve et al. A collection of breast cancer cell lines for the study of functionally distinct cancer subtypes. Cancer Cell. 2006; Prat A et al. Phenotypic and molecular characterization of the claudin-low intrinsic subtype of breast cancer. Breast Cancer Research. 2010; Semir Vranic ZG, Zhao-Yi Wang. Update on the molecular profile of the MDA-MB-453 cell line as a model for apocrine breast carcinoma studies. Oncology Letters. 2011; Holliday DL, Speirs V. Choosing the right cell line for breast cancer research. Breast Cancer Research. 2011.

^2^Culture medium and FCS were obtained from Life Technologies (Paisley, UK).

^3^Nash et al. Development and characterisation of a 3D multi-cellular in vitro model of normal human breast: a tool for cancer initiation studies. Oncotarget. 2015

All cells were maintained at 37ºC with 5% CO_2_.
